# Surgical Management and Its Impact on Adjuvant Treatment in Recurrent Ipsilateral Breast Cancer: A Retrospective Cohort Study

**DOI:** 10.3390/jcm13175142

**Published:** 2024-08-29

**Authors:** Ines Torras, Isaac Cebrecos, Helena Castillo, Laura Rodríguez, Pablo Zaragoza-Ballester, Carla Sitges, Ignacio Loinaz, Marta Garcia, Meritxell Molla, Sergi Vidal-Sicart, Eduard Mension

**Affiliations:** 1 Department of Obstetrics and Gynecology and Neonatology, Hospital Clinic of Barcelona, 08036 Barcelona, Spain; itorras@clinic.cat (I.T.);; 2 Faculty of Medicine, University of Barcelona, 08007 Barcelona, Spain; 3 Department of Nuclear Medicine, Hospital Clinic of Barcelona, 08036 Barcelona, Spain; 4 Department of Nuclear Medicine, Central University Hospital of Asturias, 33011 Oviedo, Spain; 5 Department of Nuclear Medicine, Hospital 12 de Octubre, 28041 Madrid, Spain; 6 Department of Radiology, Hospital Clinic of Barcelona, 08036 Barcelona, Spain; 7 Translational Genomics and Targeted Therapies in Solid Tumors Group, August Pi i Sunyer Biomedical Research Institute (IDIBAPS), 08036 Barcelona, Spain; 8 Department of Radiation Oncology, Hospital Clinic of Barcelona, 08036 Barcelona, Spain; 9 Diagnosis and Therapy in Oncology Group, August Pi i Sunyer Biomedical Research Institute (IDIBAPS), 08036 Barcelona, Spain

**Keywords:** axillary lymph node dissection, breast cancer, breast cancer recurrence, sentinel lymph-node

## Abstract

**Background:** Breast cancer (BC) recurrence, defined as the reappearance of cancer in the ipsilateral breast after primary treatment, poses significant challenges in clinical management. Despite advances in treatment, recurrence rates persist, ranging from 0.6 to 1.5% annually, reaching 10–15% at 20 years. This study aims to analyze the surgical and oncological characteristics of patients with BC recurrence. **Methods:** This retrospective study includes 56 patients diagnosed with recurrent BC between October 2018 and April 2022. Data were collected from a prospectively maintained surgical database. A descriptive analysis was performed on the initial BC, and the recurrence, including surgical complications, was classified using the Clavien–Dindo system. The success rates of selective sentinel lymph node (SLN) biopsies and aberrant drainages were assessed based on previous surgeries. **Results**: The cohort included 55 females and 1 male, with a median age of 65.3 years. The mean time to BC recurrence was 11.5 years. Among them, 26.8% underwent breast-conserving surgery, 41.1% had a mastectomy, 21.4% had a mastectomy with reconstruction, and 10.7% had an excision over a previous mastectomy. An SLN biopsy was performed in 78.6% of cases, with higher success rates in those without a previous axillary lymph node dissection (85.7% vs. 63.2%). Aberrant drainage was more frequent in patients with a previous ALND (44.4% vs. 20%). The median follow-up was 41.3 months, with 10.7% experiencing a second recurrence. **Conclusions:** Repeat breast-conserving surgery with re-irradiation for ipsilateral recurrence is feasible and does not significantly increase complications. SLN biopsy is valuable for restaging and tailoring adjuvant therapies, with ALND not being necessary if re-SLN biopsy shows no drainage. The management of aberrant drainage remains controversial.

## 1. Introduction

Breast cancer (BC) recurrence, defined as the reappearance of BC in the ipsilateral breast following primary treatment, presents significant challenges in clinical management and patient outcomes. Despite advancements in treatment modalities, the risk of recurrence persists, with annual rates ranging from 0.6 to 1.5% [1,2], escalating to 10–15% at 20 years [3]. The risk is higher during the first two years post-treatment, occurring more frequently in triple-negative patients [4]. In contrast, recurrences after a 20-year follow-up usually appear in patients with positive hormone receptors. 

Most patients with isolated ipsilateral BC recurrence receive a mastectomy as a surgical treatment [5]. Although for many a completion mastectomy may feel the safest and hence most desirable option, others may be very keen to consider further breast conserving surgery (BCS). It is crucial to consider the psychological impact of these choices and discuss the patient’s preferences to ensure that the surgical approach aligns with their values and emotional well-being. Some retrospective studies appear to prove the oncologic safety of repeat BCS, demonstrating comparable survival outcomes to those patients undergoing a mastectomy [6,7,8,9]. The Oncology/Radiation Therapy Oncology Group in 1014 trial involved 58 women and found a low recurrence rate (5%) and high survival rate in a cohort of recurrent BC patients (95%). Of the included patients, 90% avoided mastectomy, showing that partial breast re-irradiation is an effective alternative to mastectomy for treating recurrent breast cancer [10]. 

Nonetheless, immediate reconstruction after mastectomy is currently being performed more frequently. However, there is a lack of extensive literature and guidelines on its suitability, particularly considering factors such as skin and tissue quality when previous adjuvant radiation therapy has been administered. Patients undergoing reconstruction can choose between implant-based and autologous reconstruction options. Complication rates tend to be higher with implant reconstruction compared to autologous reconstruction for these patients.

On the other hand, axillary staging continues to be crucial for BC management for both prognostication and local treatment planning. Most patients with recurrent BC have undergone previous axillary surgery, either with a sentinel lymph node (SLN) biopsy or axillary lymph node dissection (ALND). Preoperative lymphoscintigraphy and subsequent SLN biopsy is considered to be the most reliable method for identifying lymph nodes at risk of metastasis, and has become the gold standard for regional lymphatic staging of BC. According to the National Comprehensive Cancer Network (NCCN) guidelines published in 2024, in patients with local BC after BCS who had a prior SLN biopsy, repeating the SLN biopsy may be considered, although the accuracy of the repetition is unproven [11]. Additionally, after a primary mastectomy, repeating the SLN biopsy may also be considered, although data are limited in this context. Notwithstanding, in these patients, the percentage of lymph node metastases appears to be around 17–26% [12,13]. However, there is no consensus on axillary management in cases of local recurrence.

The aim of this study is to describe the surgical and oncological characteristics of a cohort of patients who suffered BC recurrence, and to describe and compare the surgical complications of the initial and posterior surgeries. A second objective was to evaluate the success rates of performing a selective SLN biopsy and aberrant drainage by extension of the previous surgery.

## 2. Materials and Methods

This is a retrospective study performed through a prospectively maintained surgical database including a sample of 56 patients all diagnosed with recurrent BC. This study adheres to the STROBE (Strengthening the Reporting of Observational Studies in Epidemiology) guidelines to ensure the quality of the reporting.

### 2.1. Patient Selection

This study was approved by the local Ethical Committee Comité de Ética de la Investigación con medicamentos del Hospital Clínic de Barcelona, according to the appropriate regulations (HCB/2022/0791, approved on 10 November 2022). Data collection followed the principles outlined in the Declaration of Helsinki. Given the retrospective nature of this study, informed consent was obtained from all patients at the time of their inclusion in the surgical database. Patients were informed about the potential use of their anonymized data for future research purposes, including studies like this one. The consent process included a thorough explanation of the study’s objectives, the type of data to be collected, and the measures in place to protect patient confidentiality. Patients were given the opportunity to ask questions and were assured that their participation was voluntary, with the option to withdraw at any time without affecting their medical care.

We extracted data and included all BC patients (including both invasive and “in situ” breast carcinoma) who were diagnosed with ipsilateral recurrent BC from January 2018 to December 2023. Patients with distant metastases at diagnosis, those who received neoadjuvant chemotherapy before the initial surgery, or those with bilateral BC were excluded to minimize potential confounders. 

To minimize selection bias, all eligible patients meeting the inclusion criteria were consecutively included. Potential confounding factors, such as age, comorbidities, and treatment modalities, were adjusted in the statistical analysis. Efforts were made to ensure data completeness and accuracy through regular audits of the database.

The sample size was determined by the number of patients meeting the inclusion criteria during the study period. Although no formal power calculation was performed due to the exploratory nature of the study, the sample size was deemed to be sufficient for descriptive analysis and for identifying potential trends in outcomes.

A descriptive analysis of the surgical and oncological characteristics of the initial BC and the BC recurrence was performed. The surgical complications were classified according to the Clavien–Dindo system. The success rates of performing a selective SLN biopsy and aberrant drainage were assessed by previous axillary surgery extension.

Axillary involvement suspicion was ruled out using ultrasound or magnetic resonance imaging (MRI), along with the assessment of distant metastasis through standard staging studies (bone scintigraphy/computed tomography of the thorax and abdomen). Lymphoscintigraphy was performed before surgery.

The surgical technique for the breast was decided by a multidisciplinary committee based on patient preferences, previous treatments, inclusion criteria for re-irradiation, and genetic results. In cases of BCS, the lesion was localized using a radioactive iodine seed. In the case of mastectomy, the option of immediate reconstruction was offered, with an assessment using plastic surgery to determine the most suitable technique based on tissue quality.

During surgery, SLNs were identified using a gamma probe with assistance from the nuclear medicine service. Between one and three SLNs were removed when drainage was in the ipsilateral axilla. If there was exclusive aberrant drainage, a selective biopsy of the aberrant SLN was performed, whether in the contralateral axilla or the internal mammary chain. SLN was not indicated if the drainage was supraclavicular. The drainage pathway was identified through preoperative lymphoscintigraphy. It was then located in the operating room using a gamma probe, followed by a skin incision over the marked site, and then the node is excised. The SLNs were sent to Pathology for intraoperative or deferred examination using the One-Step Nucleic Acid Amplification (OSNA) method or conventional techniques. Positivity for the SLN was defined according to the TNM classification and OSNA criteria. In cases where radiotracer drainage did not occur and there was no previous ALND, the surgeon intraoperatively assessed the possibility of performing a level I ALND. In cases where a lymphadenectomy had already been performed, no further axillary surgery was performed.

A wound care visit with a specialized BC unit nurse was scheduled for the week after surgery to assess postoperative complications, and an outpatient consultation with Gynecology was scheduled for 3 weeks after surgery for further evaluation. Adjuvant treatment was administered according to the consensus of the hospital’s multidisciplinary committee, following standard practice. In cases where re-irradiation was proposed, a partial breast re-irradiation was performed. Follow-up was conducted in outpatient consultations with Gynecology/Medical Oncology/Radiation Oncology, with physical examination every 4–6 months and annual mammography and ultrasound. The surgical complications were classified according to the Clavien–Dindo system: Grade I includes minor complications that do not require specific treatment, while Grade II involves pharmacological treatments. Grades III and IV require surgical interventions or intensive care, with Grade IIIa not requiring general anesthesia and Grade IIIb requiring it. Grade IV refers to life-threatening complications, and Grade V results in patient death [14]. The presence of lymphedema or arm functional limitation was confirmed during a visit with a rehabilitation physician.

### 2.2. Statistical Analysis

Continuous variables were presented with the mean, standard deviation, median, and range. Continuous variables were compared using the independent- or paired-samples T-test and presented as mean ± standard deviation. Contingency tables were assessed using Fisher’s exact test and a Chi-squared test. A *p*-value less than 0.05 was considered to be statistically significant for all analyses. Missing data were managed using a complete case analysis approach. The statistical analysis was conducted using Software for Statistics and Data Science version 15.1 (STATA, College Station, TX, USA: StataCorp LLC).

## 3. Results

This study included a cohort of 56 patients, comprising 55 females and 1 male, all diagnosed with recurrent BC between January 2018 and December 2023. The median age of the patients at the time of recurrence diagnosis was 65.3 years (±10.5 years). The oncological characteristics of the initial BC and its recurrence are described in Table 1.

The median time from the first neoplasm to the diagnosis of ipsilateral recurrence was 11.5 years (±7.5 years). The initial diagnosis was made between 1994 and 2016. Regarding the location of the ipsilateral recurrence, 20 were in the right breast and were in 36 in the left breast. In 53.6% of the patients (30/56), the recurrence occurred in the same quadrant of the breast. Among the patients, 50 out of 56 (89%) had previous BCS, while 6 out of 56 (11%) had undergone a mastectomy. Regarding axillary surgery, 20 out of 56 (36%) had no prior axillary surgery, 17 out of 56 (30%) had a prior SLN biopsy, and 19 out of 56 (34%) had a prior ALND. Additionally, 49 out of 56 patients (88%) had received prior radiation therapy. A description of the surgical treatment received for the first diagnosis and ipsilateral local recurrence is provided in Table 2.

Of the patients who underwent mastectomy, 12 received immediate reconstruction. The techniques used for immediate reconstruction included seven latissimus dorsi flaps, one deep inferior epigastric perforator (DIEP) flap, two tissue expanders, and two definitive implants.

Genetic testing was conducted in cases that met the criteria for genetic study, with alterations found in six patients (10.7%). The mutations identified were the following: one pathogenic variant in BRCA1, one pathogenic variant in BRCA2, one patient with pathogenic variants in CDKN2A and MC1R, one patient with a pathogenic variant in CHEK2, one variant of uncertain significance (VUS) in BRCA1, and another VUS in BRCA2.

The overall SLN detection rate was 44 out of 56 (79%). Table 3 and Table 4 describe the drainage rates according to previous surgeries. Nodal disease was detected in the SLNs of six patients (13.6%), of which two were macrometastases, four were micrometastases, and one was an isolated tumor cell (ITC).

Aberrant drainage occurred in fifteen patients (26.7%), specifically in the contralateral axilla (ten patients), internal mammary ipsilateral (eight patients), intramammary (three patients), and supraclavicular ipsilateral (two patients). No patients exhibited drainage of the contralateral internal mammary or contralateral supraclavicular nodes. An SLN biopsy of aberrant chains was performed in nine patients (20.4%), five being in the contralateral axilla and four being in the ipsilateral internal mammary chain. Of the 12 patients for whom an SLN biopsy was not successfully performed, 2 patients underwent ALND, obtaining no additional metastatic nodes in the final pathology report. Table 3 and Table 4 describe the detection rate of SLNs and aberrant drainage according to previous surgeries. 

Out of 56 patients, 31 (55.36%) had no complications, 21 (37.50%) experienced Grade 1 complications, 2 (3.57%) experienced Grade 2 complications, and 2 (3.57%) had Grade 3 complications. The Grade 2 complications were surgical wound infections that required antibiotic treatment. The most serious complications were a hematoma in the mastectomy and ALND bed, which required a surgical intervention and a blood transfusion, as well as necrosis of the nipple-areola complex following breast-conserving surgery, which required local debriding.

**Figure 1:**
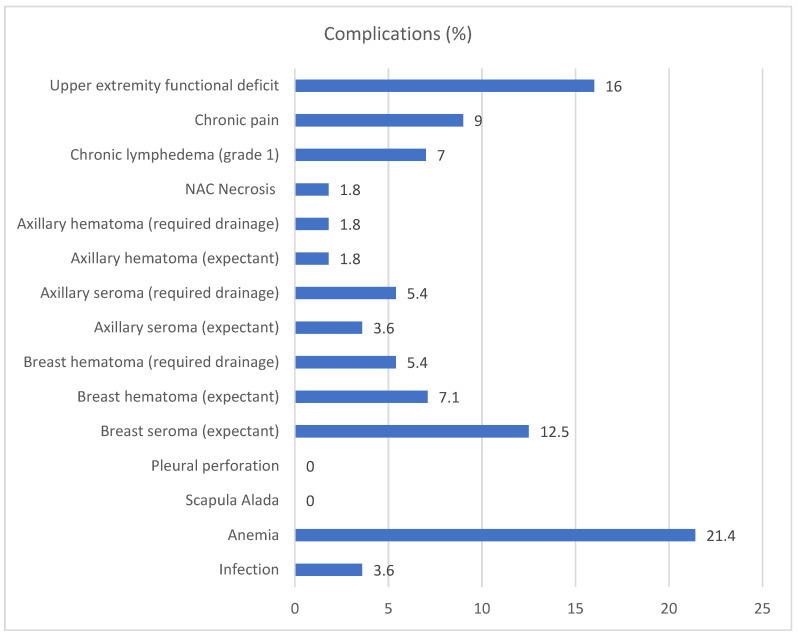
Description of the complications of breast cancer recurrence surgery, including all techniques. NAC: nipple areola complex.

In one case of internal mammary chain SLNs, moderate bleeding occurred during the intervention, and a subsequent hematoma developed in the mastectomy bed, but complete recovery was achieved with expectant management. We had no cases of pneumothorax. All of the surgical complications are described in Figure 1.

Among the patients who developed chronic lymphedema, all had undergone previous ALND. In three cases, lymphedema appeared after the second intervention where an SLN biopsy was performed. There were no cases where lymphedema occurred with only an SLN biopsy in both surgeries. In all instances, the lymphedema was classified as Grade 1.

The follow-up period after the diagnosis of recurrence was 41.3 months (±15.9 months). During the follow-up period, six patients (10.7%) experienced a second recurrence. Specifically, two local recurrences were observed in one of the patients, regional recurrence in one patient, and distant recurrence in three patients. The median time to new recurrence was 22.9 months (±10 months). Table 5 describes the oncological characteristics and previous surgeries of the patients who experienced a second recurrence.

## 4. Discussion

### 4.1. Breast Surgery: Is Mastectomy Always Needed?

The need for a mastectomy when a local recurrence of BC occurs should be evaluated on a case-by-case basis. The goal of BC surgery is to achieve the best possible oncological outcome while considering the patient’s overall health, preferences, and quality of life. If the recurrent tumor can be completely excised with clear margins without compromising cosmetic results, BCS should be considered.

One of the key factors that can limit the possibility of BCS is the history of prior radiation therapy. Traditionally, patients who have previously undergone radiation therapy were not considered to be candidates for BCS in cases of local recurrence due to concerns about cumulative toxicity. The risk of severe side effects, such as damage to the skin, soft tissue, and underlying organs, was deemed too high with additional radiation. Chen et al. [15] discouraged the use of repeat BCS following ipsilateral breast recurrence, quoting 5-year survival figures or 67% for BCS versus 78% following a salvage mastectomy—a significant difference. This difference does, however, narrow at 10 years to 57% for BCS and 62% for mastectomy. However, only 21% of the repeat BCS patients received a further course of radiotherapy. On the other hand, recent findings from the NRG Oncology/Radiation Therapy Oncology Group 1014 trial suggest that partial breast re-irradiation after a second BCS can achieve high survival rates and low recurrence rates, indicating that a second BCS is a viable alternative to mastectomy in cases with prior radiotherapy [10].

Another key factor is the potential presence of genetic mutations. According to the ASCO (American Society of Clinical Oncology) guidelines published in 2024, genetic testing is recommended in cases of second primary breast cancer [16]. This recommendation is based on the higher likelihood of underlying genetic mutations that can increase the risk of further cancer development. Identifying genetic mutations, such as BRCA1 or BRCA2, can significantly influence the surgical management plan. Patients with these mutations face a higher risk of additional BC occurrences and potentially other types of cancer. As a result, risk-reducing surgery, such as a prophylactic mastectomy, may be considered to lower the future risk of cancer.

Patients with these mutations may be counseled on the benefits and risks of undergoing a bilateral mastectomy, even if only one breast is affected, to reduce the risk of future breast cancer development. However, recent evidence indicates that bilateral mastectomy does not necessarily increase overall survival in these patients [17]. The decision-making process must involve a thorough discussion with the patient, considering their personal risk tolerance, family history, and the potential psychological impact of various surgical options.

### 4.2. Axillar Surgery: SLN Detection Rate and Aberrant Drainage

The detection rate of re-SLN, described in retrospective studies, varies between 53 and 80%, being lower in cases with prior ALND, whereas aberrant drainage has been reported around 25–50% [18,19,20]. In our series, we found an overall re-SLN detection rate of 78.6% and a higher aberrant drainage rate after previous ALND (44.4% vs. 20%, *p* = 0.06).

The most common regional drainage sites outside the ipsilateral axilla are the internal mammary region, supra/infraclavicular, and interpectoral, as well as the contralateral axilla. Besides axillary dissection, previous radiation can also influence the drainage pattern.

The removal of lymph nodes in the internal mammary chain was always more challenging due to the small size of the internal mammary nodes and the inherent difficulties of its anatomical location. In a randomized prospective study [21], an SLN biopsy in the internal mammary chain showed complete concordance between the histological status of the axillary and internal mammary SLNs. The main argument for performing an SLN biopsy in the internal mammary chain is that involvement in this location changes the patient’s staging and the adjuvant chemoradiotherapy treatment following surgery. 

### 4.3. Complications

Due to less surgical morbidity, BCS generally involves a quicker recovery and fewer postoperative complications compared to mastectomy, which can be an important factor for some patients. 

Regarding reconstruction, complication rates tend to be higher with implant reconstruction compared to autologous reconstruction for these patients [22]. Several single-institution studies have shown that implant reconstruction is feasible in this context [23,24]. In a study by Cordeiro et al. [24], early complications were reported in 29.7% of 121 patients with prior BCS and radiation, compared to 15.5% (*p* < 0.001) in 1578 patients without a history of breast surgery. In another study [25], patients who had received prior irradiation had a significantly higher risk of developing a grade III–IV capsular contracture (relative risk 3.75, *p* = 0.02) and were more likely to require autologous salvage reconstruction (relative risk 10.4, *p* = 0.02).

By providing a less-invasive method for assessing lymph node involvement, SLN biopsy helps in reducing treatment-related morbidity compared to ALND [26]. This reduction in morbidity can improve overall patient outcomes and quality of life, making patients better able to tolerate and complete their adjuvant therapy regimens.

Internal mammary node biopsy can cause pneumothorax as a side complication. The literature describes an incidence rate between 4.6 and 8% [27], although generally there is rapid recovery without additional hospitalization time.

### 4.4. Adjuvant Treatment

Recent studies explore the feasibility and safety of re-irradiation [10]. Moreover, some authors have assessed the outcomes of a second BCS with brachytherapy-based accelerated Partial Breast Irradiation (APBrI), confirming excellent oncological results, describing five-year disease-free and overall survival rates of 89% (95% CI, 86–93%) and 91% (95% CI, 88–94%), respectively [28]. Advances in radiation techniques and a better understanding of dose management have opened the door to reconsidering re-irradiation as a viable option.

The evidence on adjuvant systemic treatments is limited. The CALOR study [29], a randomized clinical trial, stands out as it evaluates the efficacy of chemotherapy in patients with locoregional BC recurrence, categorizing them based on hormone receptor status. It indicates that, at present, chemotherapy offers the best prospect of prolonged disease-free survival (DFS) in patients with ER-negative first ipsilateral locoregional recurrence, whereas adding chemotherapy to endocrine therapy seems to offer no benefit to patients with ER-positive.

On the other hand, the results of SLN can lead to modifications in adjuvant therapy regimens. For instance, if the SLN are found to be positive, it may prompt the use of more aggressive systemic therapies to address potential micrometastatic disease, or additional radiation to the axillary or supraclavicular nodes may be warranted. Conversely, negative SLN results might allow for more localized radiation treatment, sparing patients from broader radiation exposure and its associated side effects. The results of SLN biopsy can also be used to adapt follow-up and surveillance strategies. Patients with negative SLNs might have a different follow-up regimen compared to those with positive nodes, allowing for the more personalized and effective monitoring of potential future recurrences.

### 4.5. Recurrence and Survival

Patients with ipsilateral recurrent BC have 3.4–4.6 fore-hold BC-related death increased risk compared with patients who do not [30]. In a recent study with 113 patients and a median follow-up of 2.5 years, the recurrence rate for patients who underwent mastectomy was 5%, while it was 16% in the repeat BCS group [31]. However, among patients who underwent repeat BCS with re-irradiation, the risk was comparable to the mastectomy group, with only one patient developing a second recurrence (8%).

In multiple retrospective studies where ALND was omitted in cases of negative re-SLNs, no axillary recurrence was observed after a median follow-up of 27 months (range: 15–46.9 months) [32,33,34,35]. In a recent retrospective study comparing axillary surgery (SLN biopsy/ALND) versus no axillary surgery in cases of ipsilateral recurrence after BCS, it was observed that surgical axillary staging was associated with better survival [36].

### 4.6. Limitations and Future Direction

This study presents limitations. The small sample size and the retrospective nature of the data reduces the generalizability of the findings and limits the statistical power to detect significant differences or outcomes. Additionally, this study was conducted at a single institution, which may affect the external validity of the results. Another important limitation is the inability to establish causal relationships due to the observational and retrospective design of the study. While associations between surgical interventions and outcomes can be identified, it is challenging to determine whether these interventions directly cause the observed outcomes or if other confounding factors are at play. Further prospective multicenter studies with larger sample sizes are needed to confirm these findings and provide more robust evidence.

The novelty of this study lies in its detailed analysis of the type of surgery performed and the associated complications in recurrent breast cancer cases. Additionally, it introduces new insights into the success rates of SLN biopsy and the management of aberrant drainage patterns.

In light of the findings, future research should explore advanced treatment options, such as the integration of personalized medicine approaches in the management of recurrent breast cancer. Specifically, studies could investigate the efficacy of combining SLN biopsy with emerging targeted therapies to enhance treatment outcomes. Additionally, exploring the role of novel radiotherapy techniques in conjunction with re-irradiation could provide valuable insights into minimizing complications while maximizing therapeutic efficacy. 

The MARECA study [37], a prospective multicenter cohort study, represents a significant step forward in understanding the management of breast cancer locoregional recurrence. Its findings will likely provide crucial insights that could influence future surgical strategies and treatment protocols.

These advancements may offer new avenues for improving patient prognosis and quality of life.

## 5. Conclusions

The surgical management of BC recurrence entails different challenges; nonetheless, repeat breast-conserving surgery with re-irradiation for ipsilateral recurrence is feasible and does not significantly increase complications. Immediate reconstruction should be considered when undergoing a mastectomy to improve the cosmetic and psychological outcomes for the patients.

On the other hand, our study supports the use of a sentinel lymph node biopsy in the re-evaluation of patients with ipsilateral breast cancer recurrence, emphasizing its value in restaging and tailoring adjuvant therapies, meaning that axillary lymph node dissection is not necessary if re-performed sentinel lymph node biopsy shows no drainage. The management of aberrant drainage patterns remains controversial. 

Future research should continue to explore the long-term outcomes of these approaches and refine guidelines to improve the management of recurrent breast cancer.

## Figures and Tables

**Table 1:** Description of the oncological characteristics and treatments received for the first diagnosis and ipsilateral local recurrence.

Oncological Characteristics	Initial Diagnosis	Local Recurrence
Size of tumor	17.3 mm (range 2–66)	18.7 mm (range 4–77)
Focality		
Unifocal	52 (92.8%)	47 (83.9%)
Multifocal	4 (7.2%)	6 (10.7%)
Multicentric	0 (0%)	3 (5.4%)
Histological subtype		
DCIS	14 (25%)	6 (10.7%)
IDC	39 (69.7%)	43 (76.8%)
ILC	3 (5.3%)	4 (7.1%)
Other	0 (0%)	3 (5.4%)
IHQ subtype (only infiltrating)		
Luminal (HR+/Her2 negative)	34 (83.3%)	39 (76.5%)
HR+/Her2 positive	0 (0%)	2 (3.9%)
HER2 positive	2 (4.8%)	3 (5.9%)
TN	5 (11.9%)	6 (11.7%)
Initial TNM staging (T)		
Tx	6 (10.7%)	0 (0%)
Tis	14 (25%)	5 (8.9%)
T1mi	0	3 (5.4%)
T1a	0	5 (8.9%)
T1b	8 (14.3%)	10 (17.9%)
T1c	14 (25%)	23 (41%)
T2	12 (21.4%)	8 (14.2%)
T3	2 (3.6%)	2 (3.6%)
Initial TNM staging (N)		
Nx	12 (21.4%)	7 (12.5%)
N0	26 (46.4%)	43 (7.7%)
N0 (itc)	3 (5.4%)	1 (1.7%)
N1	15 (26.8%)	5 (8.9%)
Neoadjuvant therapy	8 (14.3%)	11 (19.6%)
Adjuvant therapy		
Radiotherapy	49 (87.5%)	21 (37.5%)
Endocrine therapy	31 (55.4%)	43 (76.8%)
Chemotherapy	19 (33.9%)	12 (21.4%)
Targeted therapy	2 (3.6%)	5 (8.9%)

DCIS: ductal carcinoma in situ, IDC: invasive ductal carcinoma, ILC: invasive lobular carcinoma, IHQ: immunohistochemistry, HR: hormonal receptors, TN: triple negative.

**Table 2:** Description of the surgical treatment received for the first diagnosis and ipsilateral local recurrence.

Surgical Techniques	Initial Diagnosis	Local Recurrence
Breast surgery		
BCS	50 (89.3%)	15 (26.8%)
Mastectomy	4 (7.1%)	23 (41.1%)
Mastectomy with reconstruction	2 (3.6%)	12 (21.4%)
Excision over previous mastectomy	0 (0%)	6 (10.7%)
Type of reconstruction		
Implant based reconstruction	1 (50%)	4 (33.3%)
Autologous reconstruction	1 (50%)	8 (66.7%)
SLN biopsy		
Yes	34 (60.7%)	44 (78.6%)
No	22 (39.3%)	12 (21.4%)
Number SLN	1.9 (range 1–5)	Mean 1.7 (range 1–5)
SLN positive		
Yes	10 (29.4%)	6 (13.6%)
No	24 (70.6%)	38 (86.3%)
ALND		
Yes	19 (33.9%)	7 (12.5%)
No	35 (62.5%)	49 (87.5%)
Total positive nodes	2 (range 1–7)	1 (range 1–1)
Total nodes	7.4 (range 1–26)	2.95 (range 1–11)

BCS: breast-conserving surgery, SLN: sentinel lymph node, ALND: axillary lymph node dissection.

**Table 3:** Description of the success rates of performing selective SLN biopsy based on previous surgery.

	SLN Harvesting Rate	
Previous breast surgery		
Breast conserving surgery	37 (75.5%)	
Mastectomy	4 (100%)	
Mastectomy with reconstruction	2 (100%)	*p* = 0.25
Previous SLN biopsy		
Yes	26 (76.5%)	
No	16 (80%)	*p =* 0.5
Previous ALND		
Yes	12 (63.2%)	
No	30 (85.7%)	*p =* 0.06

SLN: sentinel lymph node. ALND: axillary lymph node dissection

**Table 4:** Description of aberrant drainage rate based on previous surgery.

	Aberrant Drainage	
Breast surgery		
Breast-conserving surgery	12 (24.5%)	
Mastectomy	2 (50%)	
Mastectomy with reconstruction	1 (50%)	*p* = 0.2
Previous SLN biopsy		
Yes	8 (53.3%)	
No	7 (46.7%)	*p* = 0.5
Previous ALND		
Yes	8 (44.4%)	
No	7 (20%)	*p* = 0.06

SLN: sentinel lymph node, ALND: axillary lymph node dissection.

**Table 5:** Description of the oncological characteristics and previous surgeries of patients with a new recurrence during follow-up.

Oncological Characteristics	Previous Surgery	Time to Recurrence	Recurrence
pT1c pN1mi Luminal B-like	Mastectomy + SLN biopsy	28 months	Distance recurrence (hepatic metastasis)
pT1c pNx Luminal B-like	Mastectomy (no SLN biopsy, previous ALND)	3 months	Distance recurrence (pulmonary metastasis)
pT2 pN0 Luminal B-like VUS BRIP1	Mastectomy + SLN biopsy	3 months	Distance recurrence (bone metastasis)
pT1c pN0 Luminal B-like (secretor)	Excision over previous mastectomy + SLN biopsy	14.9 months	Axillary recurrence
pT1c pN0 Luminal A-like	Excision over previous mastectomy + SLN biopsy	24 months	Local recurrence
pT1c pN0 Luminal B like Refused chemotherapy	BCS + SLN biopsy	63 months	Local recurrence

SLN: sentinel lymph node, ALND: axillary lymph node dissection.

## Data Availability

The datasets presented in this article are not readily available because due to time limitations. Requests to access the datasets should be directed to mension@clinic.cat.
